# Does pulmonary surfactant generally affect antimicrobial activity?

**DOI:** 10.1186/2050-6511-13-S1-A62

**Published:** 2012-09-17

**Authors:** Richard Schwameis, Sabine Strommer, Markus Zeitlinger, Robert Sauermann

**Affiliations:** 1 Department of Clinical Pharmacology, Section of Clinical Pharmacokinetics, Pharmacogenetics and Imaging, Medical University of Vienna, 1090 Vienna, Austria

## Background

Activity of antimicrobial agents may be affected by pulmonary surfactant. Notably, it was reported that the clinical efficacy of daptomycin is significantly impaired in pneumonia in spite of bacterial susceptibility *in vitro*. This study set out to assess the impact of pulmonary surfactant *in vitro* on bacterial killing of other antibiotics used for treatment of pneumonia.

## Methods

Time-kill curves of daptomycin, doripenem, linezolid, moxifloxacin, and tigecycline were determined for *Staphylococcus aureus* ATCC 29213 and of colistin, doripenem and moxifloxacin for a clinical isolate of *Pseudomonas aeruginosa* at concentrations above or equal to the respective MICs. All experiments were performed over 24 h in Mueller-Hinton broth (MHB) and in MHB enriched with porcine surfactant at a concentration of 1 mg/mL (MHB_surf_).

## Results

As expected, daptomycin was not bactericidal in presence of surfactant at concentrations up to 64 times the MIC. In MHB_surf_ a higher concentration of moxifloxacin (16x) was needed than in MHB (2x MIC) to achieve sustained bacterial killing of *S. aureus*. In contrast, killing of *P. aeruginosa* by moxifloxacin was not affected by surfactant. A slightly higher concentration of doripenem (8x) was needed in MHB_surf_ to achieve sustained antimicrobial killing against *S. aureus* than in MHB (4x MIC). However, killing was faster in MHB_surf_. Similarly, initial killing of *S. aureus* by tigecycline was faster in MHB_surf_ than in MHB while after 24 hours no difference in bacterial counts was observed between MHB and MHB_surf_. For linezolid no significant effects were observed by adding surfactant. Likewise, surfactant had no significant influence on the activity of colistin and doripenem against *P. aeruginosa*.

## Conclusions

The activity of moxifloxacin against *S. aureus* was reduced *in vitro* by addition of surfactant whereas this effect could not be observed against *P. aeruginosa*. Interestingly, antimicrobial killing by several antibiotics of Gram-positive *S. aureus*, but not of Gram-negative *P. aeruginosa* tended to be faster in presence of surfactant. Thus, apart from daptomycin, pulmonary surfactant is also capable of influencing the bacterial killing kinetics of several other antibiotics. The clinical relevance of these *in vitro* findings for pneumonia patients is currently unclear, and should be carefully evaluated.

